# Information Seeking Regarding Tobacco and Lung Cancer: Effects of Seasonality

**DOI:** 10.1371/journal.pone.0117938

**Published:** 2015-03-17

**Authors:** Zhu Zhang, Xiaolong Zheng, Daniel Dajun Zeng, Scott J. Leischow

**Affiliations:** 1 The State Key Laboratory of Management and Control for Complex Systems, Institute of Automation, Chinese Academy of Sciences, Beijing, China; 2 Department of Management Information Systems, The University of Arizona, Tucson, United States of America; 3 Mayo Clinic, Scottsdale, Arizona, United States of America; University of Warwick, UNITED KINGDOM

## Abstract

This paper conducted one of the first comprehensive international Internet analyses of seasonal patterns in information seeking concerning tobacco and lung cancer. Search query data for the terms “tobacco” and “lung cancer” from January 2004 to January 2014 was collected from Google Trends. The relevant countries included the USA, Canada, the UK, Australia, and China. Two statistical approaches including periodogram and cross-correlation were applied to analyze seasonal patterns in the collected search trends and their associations. For these countries except China, four out of six cross-correlations of seasonal components of the search trends regarding tobacco were above 0.600. For these English-speaking countries, similar patterns existed in the data concerning lung cancer, and all cross-correlations between seasonal components of the search trends regarding tobacco and that regarding lung cancer were also above 0.700. Seasonal patterns widely exist in information seeking concerning tobacco and lung cancer on an international scale. The findings provide a piece of novel Internet-based evidence for the seasonality and health effects of tobacco use.

## Introduction

Tobacco is the only legal drug that causes many deaths of its users when used exactly as intended by manufacturers [1]. Tobacco smoking is associated with an increased risk of ill-health, disability, and death from noncommunicable chronic diseases (e.g., lung cancer and cardiovascular disease) as well as communicable diseases such as tuberculosis [1]. Direct tobacco smoking causes about the deaths of 5 million people across the world each year, and many of these deaths occur prematurely [1,2]. Given the serious damage of tobacco smoking, tobacco control is one of the most important global public health priorities.

Exploring temporal patterns (e.g., seasonality) of smoking behavior can significantly help us to make reasonable tobacco control regulations. For instance, a previous research suggests that findings on seasonality of smoking may have major implications for the timing of tobacco control measures and interventions [3]. Several existing empirical studies [3,4,5,6,7] have found that seasonality exists in the cigarette sales [3,6], the sales of nicotine replacement therapies [4], the initiation of smoking among adolescents [5], and the onset of youth smoking [7]. Most of these studies were conducted based on the actual and offline survey data. This kind of data collected by survey-based tobacco control surveillance is costly to obtain, and often lacks timeliness [8,9]. In addition, the survey data in the above previous studies is limited to the region of the USA. To the best of our knowledge, no comprehensive study on seasonality of tobacco use has been conducted on an international scale. The major reason may be that it is difficult to obtain suitable data across different countries for such study.

However, this situation presented above is changing. Recently, with the rapid growth of Internet, there is a large number of online search data about public health (e.g., tobacco and lung cancer etc.), and such search data suggests public interest in public health information. The timely, large-scale, public, and free Internet search query data provides a novel resource for public health researchers to obtain a better understanding of collective human behaviors. For instance, some previous studies used Web search queries to monitor infectious and noninfectious diseases such as influenza epidemics [10,11,12] and other diseases [13,14]. Tobacco control researchers have also used Internet search queries to explore issues such as studying who searched smoking cessation information and how they searched it using Web search engines [15,16], monitoring tax avoidance and smoking cessation after the increase of cigarette tax [17], and tracking the popularity of electronic cigarettes [18]. Additionally, the impact of World No Tobacco Day in Latin America was evaluated by health-related news stories and Internet search queries [8]. Since some previous studies show that the weather condition (especially temperature) may impact on the seasonal variation of smoking behavior [3,6], tobacco and lung cancer information seeking relevant to smoking may also have seasonal patterns. To complement the mentioned offline survey data, the aim of this paper is to exploit Internet search data to monitor continuous population interest in tobacco and lung cancer and study seasonal patterns of information seeking concerning tobacco and lung cancer.

## Materials and Methods

### Ethics Statement

The raw data used in this study was freely downloaded from Google Trends (http://www.google.com/trends/), a public service provided by Google Inc., which allows Internet users to examine trends of certain query terms by time, geographic location, and category. The search query trend data collected from Google Trends is already anonymized. The data doesn’t involve human subject research, clinical trials, animal research, and observational and field studies, so we don’t need to obtain ethical oversight from an Institutional Review Board (IRB) or from an appropriate data protection agency. Additionally, Google has relevant privacy policies to address ethical issues related to Google Trends. The raw datasets in this study were deposited as S1 and S2 Datasets in supporting information.

### Data collection

Due to the above advantages of Internet search queries, Google Trends was used to monitor the incidence of depression [14], smoking cessation [17], the popularity of electronic cigarettes [18], the interest in behavior change [19], and the evaluation of World No Tobacco Day [8]. Recently, it was applied to monitor non-cigarette tobacco use [20]. Besides, another study presented an external revision to Google Flu Trends for improving the prediction accuracy [21]. On Google Trends, we used “tobacco” as the search query term to collect the search trend data about tobacco in the four main English-speaking countries (i.e., the USA, the UK, Canada, and Australia). We also used the Chinese translation of “tobacco” as the query term for the trend data in China. In a similar way, the keyword “lung cancer” was used to collect the trend data regarding lung cancer (i.e., one of the main smoking-attributable diseases). The query terms used in this study were limited to all categories rather than a specific category like “health” or “shopping” on Google Trends, because the information related to the query term “tobacco” or “lung cancer” can cover several categories on Google Trends. The query terms “tobacco” and “lung cancer” were chosen since they were the most suitable to accurately represent information seekers’ interest in tobacco and lung cancer information, respectively. For instance, the information seeking interest indicated by the search terms “tobacco use” or “tobacco smoking” was narrow than that of the search term “tobacco”. Based on the direct observations on Google Trends, the search trends of the query term “cigarette” were similar to those of the query term “tobacco”. Besides, cigarette is one of tobacco products, and the concept of tobacco use is wider than cigarette smoking. Therefore, the search trends about cigarette were not considered in this article. The selection of the above Chinese query terms followed the same rule. The original data was downloaded on 27 October 2014, and the time interval covered by the data was from January 4, 2004 (the first date when the data was available) to October 25, 2014 (the end date). Then we extracted the ten-year data containing 522 weeks from the beginning to January 4, 2014 for convenience. The methodology to generate Google Trends data has been presented in [11]. Briefly, Google aggregates historical search query logs for chosen terms submitted within a given time frame and region. A relative value for a chosen query term can be calculated as the total search volume for the term divided by the total search volume within the chosen geographic region and time [19]. Then the relative value is normalized to the peak relative value over a given time period with respect to the same term. Finally, all relative query values are from 0 to 100.

### Statistical analysis

The following statistical analysis methodology was borrowed from that in our previous work [22], but the data, related analyses, and discussion were considerably extended in this study.

Periodogram (also known as spectral plot) was applied to determine whether seasonal patterns of information seeking concerning tobacco and lung cancer existed in the corresponding search trend data across different countries, respectively. A peak in a periodogram indicated that a seasonal component existed near the frequency value corresponding to the peak. When a periodogram was generated, we applied ideal pass filter on the periodogram to extract a seasonal component from the raw trend data. The frequency interval for the filtering process just covered the peak shape in the periodogram. Ideal pass filter is a data filtering tool offered by the time series tools of MATLAB R2010b. According to the user’s guide, ideal pass filter allows only the variations in a specific frequency range. “These filters are ‘ideal’ in the sense that they are not realizable; an ideal filter is non-causal and the ends of the filter amplitude are perfectly flat in the frequency domain [23].”

Moreover, to examine linear and temporal associations of seasonal components of these trend data among countries, we calculated the pairwise cross-correlations of these seasonal components. In detail, we conducted the correlation analysis to quantify how much these seasonal components were correlated and find the time shifts in the seasonalities among different countries. If there are high cross-correlations between search trends of different countries, there are common temporal patterns in the tobacco or lung cancer information seeking behavior across countries. These common patterns of information seeking may be used in the timing of international tobacco control campaigns. For instance, if there is a high cross-correlation between the tobacco search trends of the USA and Canada, and the time lag is 0, they can collaborate to start an anti-smoking campaign at the peak of tobacco information seeking, because the information seekers’ interest to tobacco information is the highest at that time. Then the trend data concerning lung cancer was also analyzed like the tobacco data. Similarly, we measured the temporal associations between seasonal components of the trend data regarding tobacco and those regarding lung cancer by cross-correlation. Note that cross-correlation was used to measure the degree of the linear relationship between two search trends at various time lags in this study. A high correlation at a specific lag might indicate that these two trends had certain same influencing factors and a time delay [23]. The periodogram, ideal pass filter, and cross-correlation were carried out by the time series tools of MATLAB [23]. For the details of these tools, see the previous study [22] and the MATLAB manual [23].

## Results

**Fig 1:**
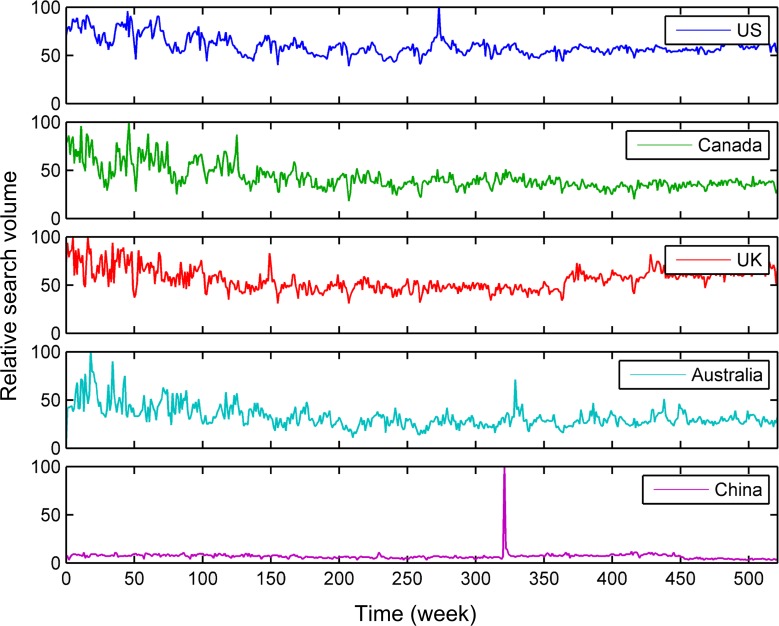
Raw search trends regarding tobacco from Google Trends. It contains the trends of the USA, Canada, the UK, Australia, and China. The time interval contains 522 weeks from January 4, 2004 to January 4, 2014.

**Fig 2:**
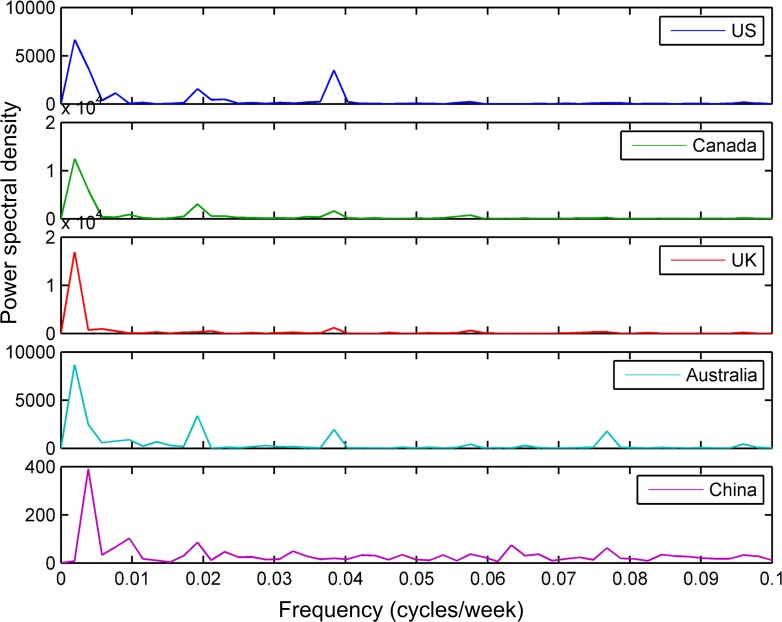
Periodograms of the tobacco-related search trends of the USA, Canada, the UK, Australia, and China. A peak in the periodogram indicates there is a seasonal component near the value corresponding to the peak.

Fig. 1 showed the time series plots of the raw trend data regarding tobacco (See S1 Fig. for the trends related to lung cancer). Note that all figures in this study were produced by the time series tools of MATLAB [23]. The horizontal axis was the time line with one week as the unit time. Fig. 2 presented the periodograms of the search trend data about tobacco (See S2 Fig. for the periodograms about lung cancer). In the periodograms, the salient peak near 0.0192 on the horizontal line indicated that each search trend had a seasonal component whose period was about one year (i.e., 1 cycle / [0.0192 cycles/week] ≈ 52 weeks). Another salient peak near 1.919×10^–3^ indicated the seasonal component whose period was the whole time interval (i.e., 1 cycle / [1.919×10^–3^ cycles/week] ≈ 521 weeks) of the search trend. In addition, the seasonal component with half-year period was indicated by the peak near 0.04. We omitted other peaks in these periodograms, because they didn’t widely exist in these periodograms.

**Fig 3:**
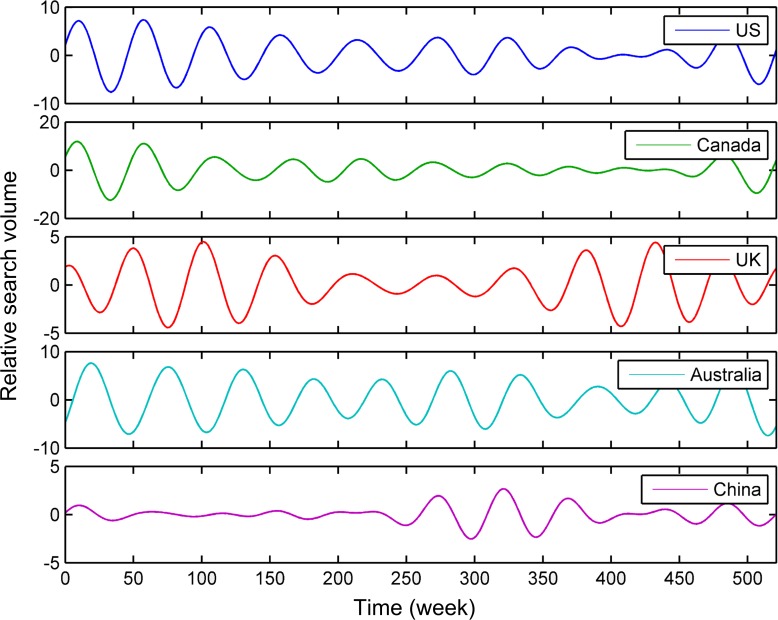
Seasonal components of the tobacco-related search trends of the USA, Canada, the UK, Australia, and China. These seasonal components are extracted by using ideal pass filter on the raw search trends.

In Fig. 2, the one-year and half-year seasonal components were more significant than other seasonal components. Since the previous study indicated the seasonality of cigarette sales in the USA had a 12-month period [3], only the one-year seasonal components were considered in detail in this paper. As illustrated in Fig. 3 and S3 Fig., the seasonal components with one-year period were extracted by using ideal pass filter on the raw trends, when we selected the frequency range from 0.015 to 0.025 in the periodograms. This frequency range only covered each peak shape near 0.0192 approximately, but didn’t cover other peak shapes in Fig. 2 and S2 Fig. For Fig. 1, S1 Fig., Fig. 3 and S3 Fig., the relative search volumes of the countries on the northern (i.e., the USA, Canada, the UK, and China) and southern (i.e., Australia) hemispheres had an approximate sinusoidal pattern. The search trend of the USA almost had the same peak and trough with that of Canada, and the peak and trough of the search trend of the UK was close to that of the USA. The trend of Australia was nearly the inversion of the UK's search trend at the former half part of the search time series. The seasonal pattern of the search trend of China was not so regular as those of the other trends.

Before the cross-correlation analyses of the trends were conducted, we used Augmented Dickey-Fuller test to detect whether the extracted seasonal components of the studied trends were stationary. The results indicated all of them were stationary (See S3 and S4 Tables for details). In all tables of this paper, each cell contained two values: a correlation coefficient and a time lag that was in the parenthesis. For each correlation coefficient in all correlation tables, the row represented the first time series, and the column represented the second time series. Each lag value indicated that the number of time steps by which the second time series was shifted relative to the first time series. The unit of the time lag was week. In all cross-correlation plots, all lags were limited to the scale from −52 to 52 due to the one-year period of the seasonal components. Each time lag in each table was selected to provide the highest correlation among the paired cross-correlation values and time lags, and the correlation tables at lag 0 were provided in supporting information (See S1 and S2 Tables). For example, in Table 1, the cross-correlation coefficient of the seasonal components related to tobacco search trends of the USA and Australia was 0.651, and (-15) meant Australia fell behind the USA 15 weeks in terms of these seasonal components. From Table 1, we could observe that for two countries on the same hemisphere, the time lags corresponding to their correlation coefficients had smaller lengths than those of two countries on the different hemisphere except the coefficients of the UK and Canada (or China). For instance, the length of the time lag corresponding to the correlation of the USA and Canada equaled 0, smaller than that of the USA and Australia (i.e., it equaled −15). As shown in Table 1, for the correlations of these English-speaking countries, four out of six were above 0.600. Besides, two out of four were above 0.500 for the correlations of China with the other countries. Therefore, we could infer that seasonal components in the tobacco-related search trends had a significant positive correlation with each other for these four English-speaking countries.

**Table 1:** The pairwise cross-correlations of seasonal components of tobacco-related search trends.

	Country				
Country	US	Canada	UK	Australia	China
**US**	1.000	0.906 (0)	0.717 (5)	0.651 (-15)	0.585 (0)
**Canada**	—	1.000	0.596 (-18)	0.788 (-14)	0.461 (-1)
**UK**	—	—	1.000	0.447 (33)	0.360 (-52)
**Australia**	—	—	—	1.000	0.581 (10)
**China**	—	—	—	—	1.000

The values in each cell contain two parts: a correlation coefficient and a time lag in the parenthesis.

The values in each cell contain two parts: a correlation coefficient and a time lag in the parenthesis.

As shown in Table 2, the time lags of countries on the same hemisphere had smaller lengths than those on the different hemisphere except the coefficients of China and the USA (or Canada). Besides, for the correlations of these English-speaking countries, five out of six were above 0.600. Besides, one out of four was above 0.500 for the correlations of China with the other countries. The observation indicated that seasonal components in the search trends about lung cancer had a significant positive correlation with each other for these four English-speaking countries.

**Table 2:** The pairwise cross-correlations of seasonal components of search trends regarding lung cancer.

	Country				
Country	US	Canada	UK	Australia	China
**US**	1.000	0.950 (1)	0.558 (5)	0.856 (12)	0.369 (51)
**Canada**	—	1.000	0.610 (4)	0.769 (-13)	0.348 (-51)
**UK**	—	—	1.000	0.612 (31)	0.719 (4)
**Australia**	—	—	—	1.000	0.419 (-31)
**China**	—	—	—	—	1.000

Table 3 summarized the cross-correlations of the seasonal components of the search trends concerning tobacco and lung cancer. All correlation coefficients except that of China in the table were above 0.600. This observation indicated that the seasonal components of the search trends regarding tobacco had a significant positive correlation with that regarding lung cancer. One possible reason is that the association between tobacco and lung cancer has become very well known among those in the health communities, perhaps many information seekers will search for tobacco and lung cancer information together.

**Table 3:** The cross-correlations between seasonal components of search trends regarding tobacco and those regarding lung cancer.

	Lung Cancer				
Query Term	US	Canada	UK	Australia	China
**Tobacco**	0.757 (-2)	0.845 (-1)	0.734 (0)	0.858 (0)	0.476 (51)

## Discussion

The first important finding of this paper is that seasonality widely exists in the Internet search trend data concerning tobacco on an international scale. The second finding is that seasonal components of the search trend data regarding tobacco have a significant positive correlation with each other across countries. These two findings complement the previous studies about smoking-related seasonality which used real-world survey data limited to the USA [3,4,5,6,7]. The third finding is that the Internet search trend data concerning lung cancer is similar to that of tobacco-related trend data in terms of seasonality. The fourth finding is that the seasonal components of the trend data regarding tobacco have a significant positive correlation with those regarding lung cancer. These findings about lung cancer provide a piece of novel Internet-based evidence for the health effect of tobacco use, although the significant correlation between smoking and lung cancer has been well-documented in related medical literature [1,24]. For the seasonality of the search trends and the peaks and troughs in the seasonal components of these trends, weather condition (especially temperature) may be one important factor of the reason, since some previous studies show that the weather condition (especially temperature) may be a factor contributing to the seasonal variation of smoking behavior [3,6]. In addition, the reason for the above mentioned time lag length may be that the similar weather condition (especially temperature) on the same hemisphere results in the similar smoking-related information seeking on the same hemisphere, based on the studies [3,6].

There are several implications in this study. First, the findings about seasonality in the Internet search trend data regarding tobacco and lung cancer provide the potential to help tobacco control professionals with the timing of tobacco control effort. Sheffer et al. demonstrated the increased participation in a statewide tobacco quitline service could be produced by launching a statewide media campaign timed to coincide with temporal smoking cessation behavioral patterns [25]. Similarly, it would be more effective for Internet users to access tobacco control information if some tobacco control campaigns are carried out at the peak period of tobacco information seeking. The reason is that information seekers’ interest in tobacco information is the highest at that time. Second, the proposed investigation methodology for seasonality in Internet search trends can be extended to other disciplines including public health, sociology, and economics. By leveraging seasonality in Internet search trends, predictive models can be easily estimated [26]. Third, besides the Internet search query data, social media such as Facebook, Twitter, YouTube [27] etc. has become a new information source for researchers of different disciplines. Therefore, exploring collective intelligence in social media with social computing approaches [28,29] and new temporal or spatio-temporal analysis methods [30,31,32] can provide a novel insight for public health research (e.g., the analysis of smoking and smoking cessation behavior).

This study has the following limitations. First, the countries involved in the search trends only include four English-speaking developed countries and a developing country (i.e., China). The other English-speaking countries such as India, South Africa, and Pakistan are excluded in this study, because they are multilingual countries and using English query terms can’t retrieve the effective and comprehensive search trends. In addition, the search trends about tobacco and lung cancer in New Zealand are not included because there are a number of missing values in the trend data. Therefore, sampling data from these countries may limit the generalizability of our findings. Second, the Internet search query data mainly reflects information seekers’ interest in information regarding search query terms. We didn’t compare the online search data with real-world data, as it was very hard for us to collect such rea-world tobacco and lung cancer data of these countries. Future work is needed to show to what extent the Internet search query data about tobacco and lung cancer can represent the real-world data about tobacco and lung cancer, if we can collect some appropriate real-world data. Additionally, more studies are required to investigate that what factors attribute to the reasons for the seasonality of the search trends and the peaks and troughs in the seasonal components of these trends. One possible explanation for seasonality may be that some tobacco-related conferences held at the same time of each year result in the much relevant information seeking on the Internet at the specific time of each year.

### Conclusions

In summary, we studied seasonal patterns in information seeking concerning tobacco and lung cancer using Internet search information. Our findings indicate that seasonal patterns widely exist in information seeking concerning tobacco and lung cancer and have close correlations with each other across countries. Exploring the emerging online information including Internet search queries and social media information can provide novel data sources and investigation insights for public health research.

## Supporting Information

S1 Fig The raw search trends on lung cancer of the USA, Canada, the UK, Australia, and China from Google Trends, from January 4, 2004 to January 4, 2014. (TIF)

S2 Fig The periodograms of search trends on lung cancer in the USA, Canada, the UK, Australia, and China. (TIF)

S3 Fig The seasonal components of search trends about lung cancer of the USA, Canada, the UK, Australia, and China. (TIF)

S4 Fig The raw search trends of the query terms “cold” and “potato” of the USA. (TIF)

S5 Fig The periodograms of search trends of the query terms “cold” and “potato” of the USA. (TIF)

S6 Fig The raw search trends of the query terms “Google” and “pizza” of the USA. (TIF)

S7 Fig The periodograms of search trends of the query terms “Google” and “pizza” of the USA. (TIF)

S1 Table The pairwise cross-correlations (at lag 0) of seasonal components of tobacco-related search trends. (DOCX)

S2 Table The pairwise cross-correlations (at lag 0) of seasonal components of search trends regarding lung cancer. (DOCX)

S3 Table Stationarity test of seasonal components of tobacco-related search trends. (DOCX)

S4 Table Stationarity test of seasonal components of search trends regarding lung cancer. (DOCX)

S1 Dataset Raw search trend data regarding tobacco and lung cancer. (ZIP)

S2 Dataset Raw search trend data of the terms “cold”, “potato”, “Google”, and “pizza”. (ZIP)
