# Supplementary figures and images for: Information Seeking Regarding Tobacco and Lung Cancer: Effects of Seasonality
Source: PLoS One. 2015 Mar 17;10(3):e0117938. doi: 10.1371/journal.pone.0117938 (PMC4364309; doi:10.1371/journal.pone.0117938)

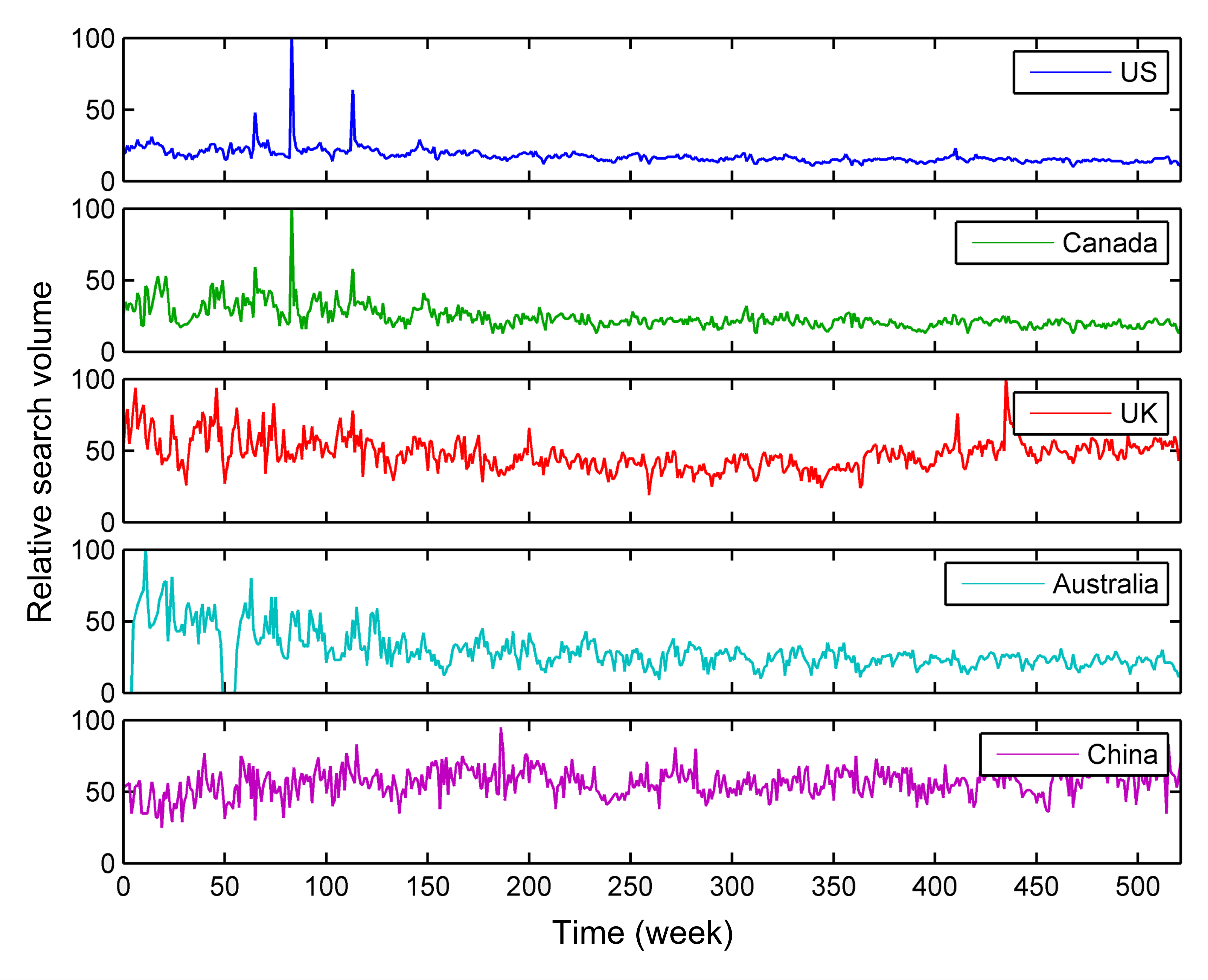

Supplement: S1 Fig — (TIF) [file pone.0117938.s001.tif]

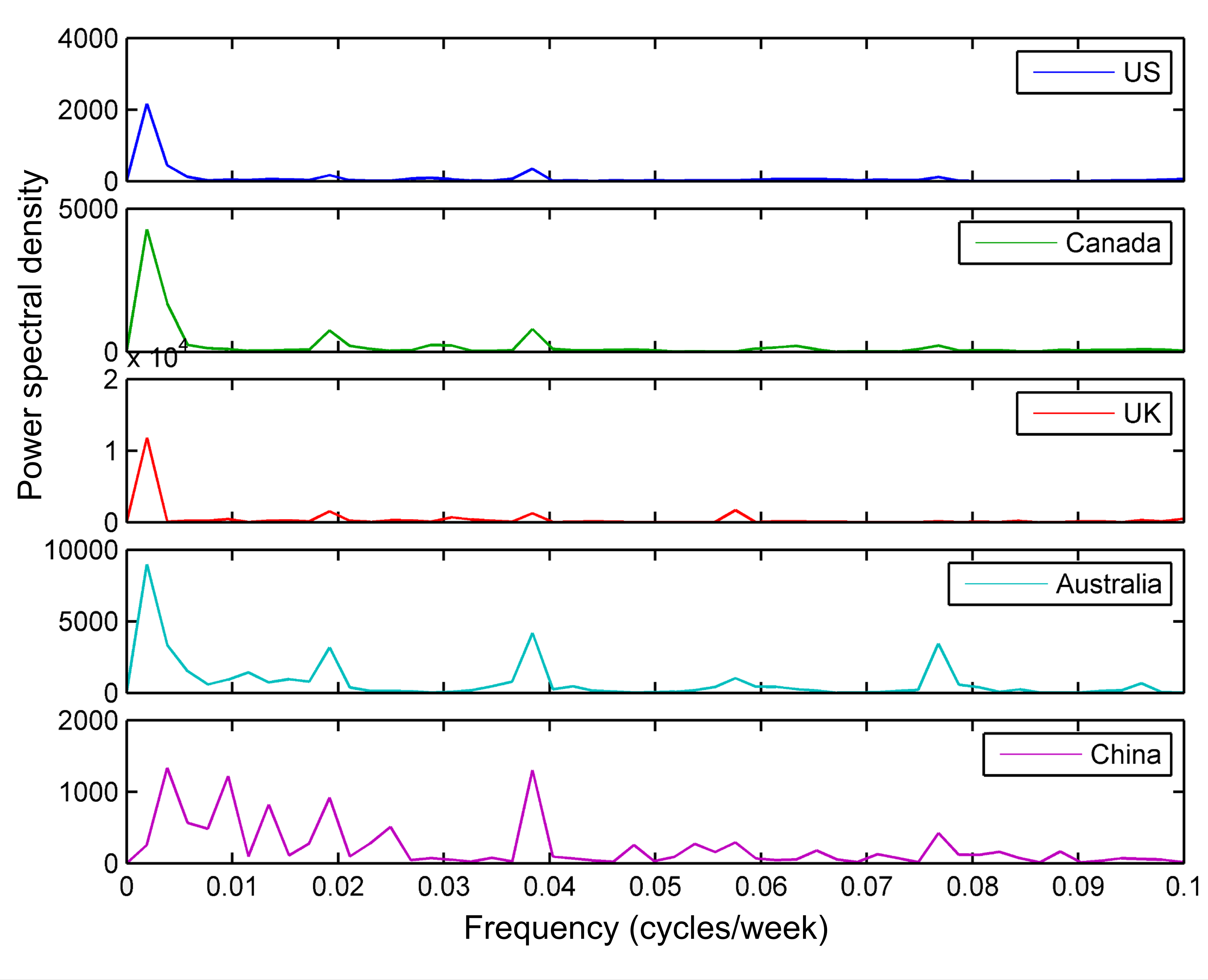

Supplement: S2 Fig — (TIF) [file pone.0117938.s002.tif]

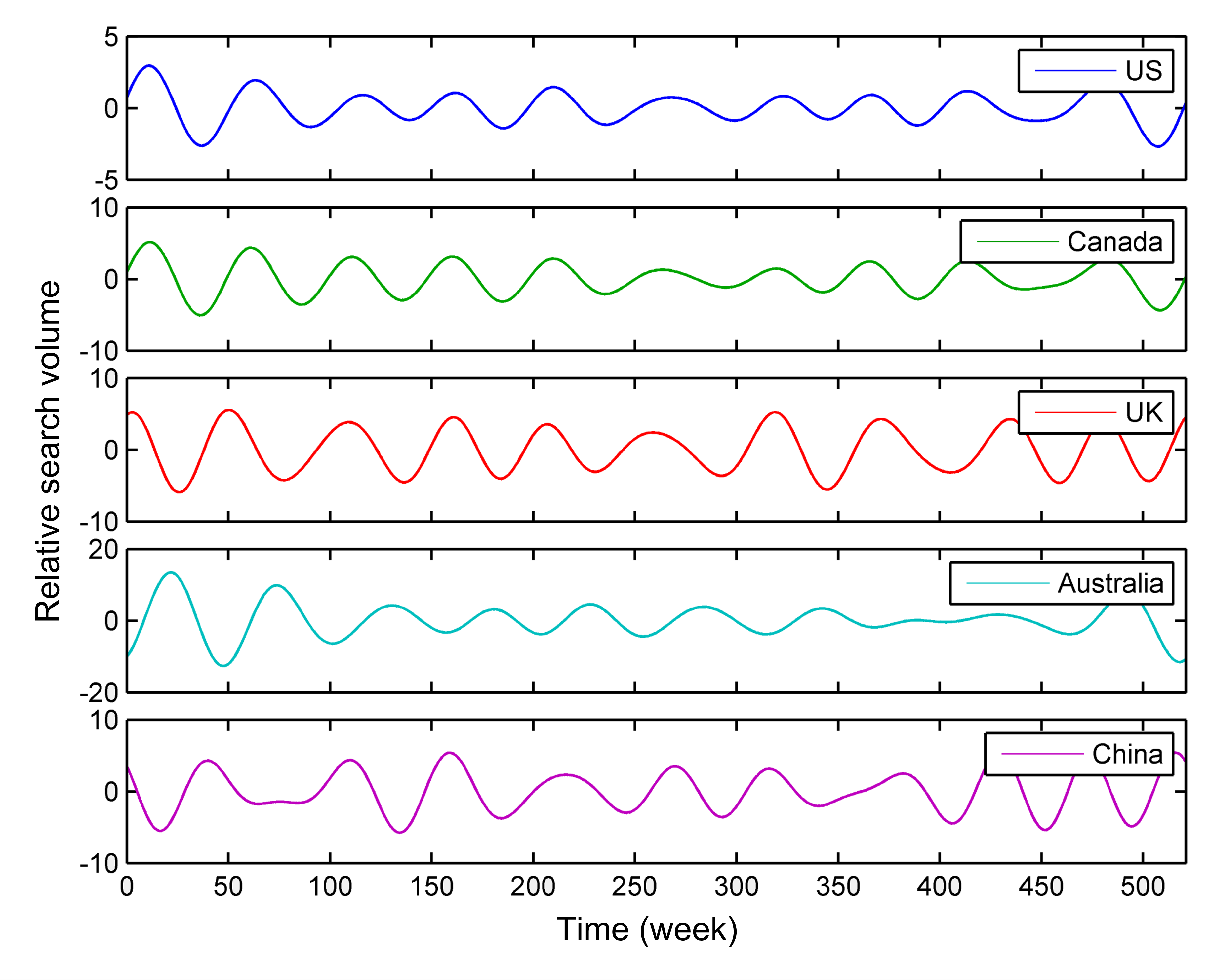

Supplement: S3 Fig — (TIF) [file pone.0117938.s003.tif]

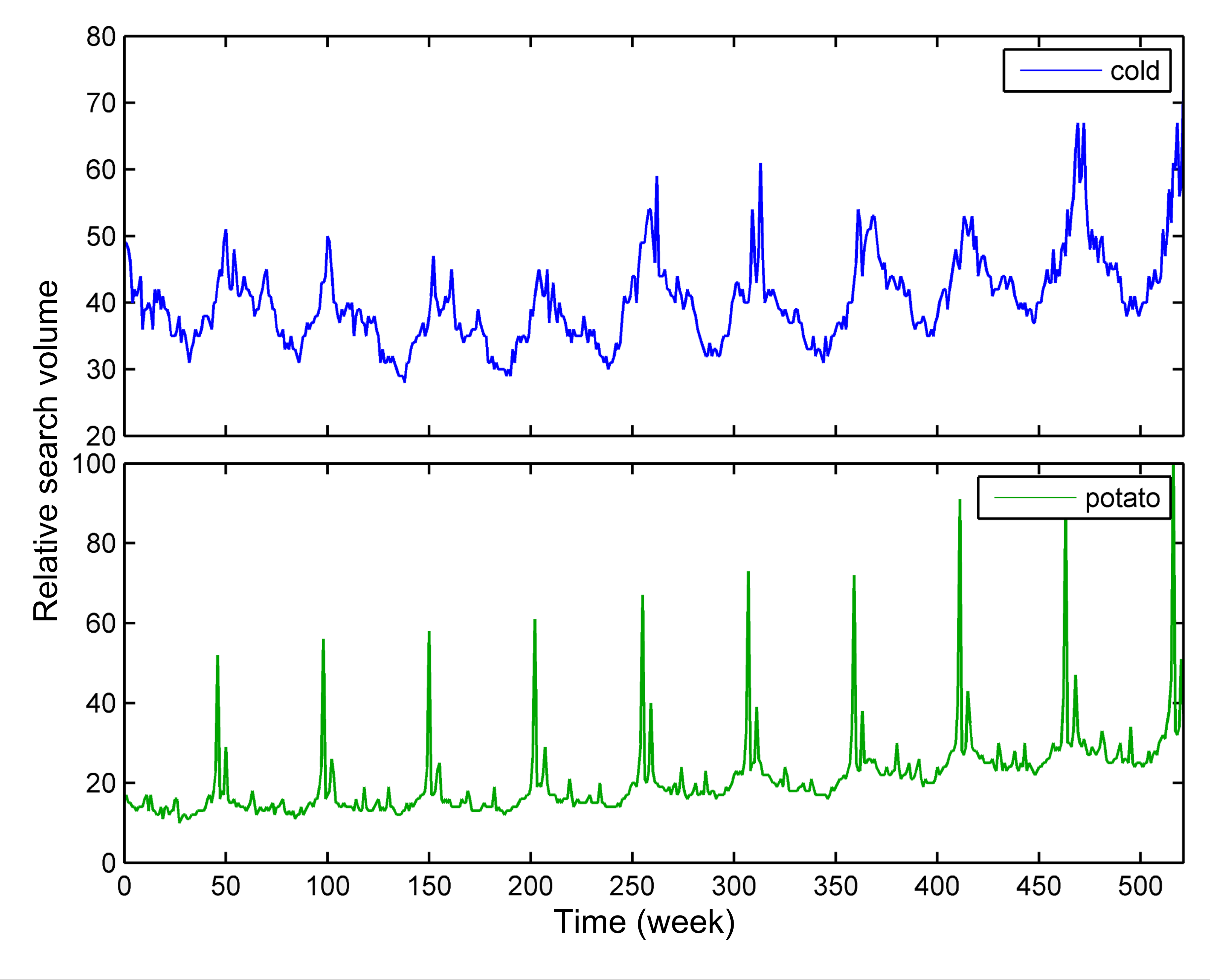

Supplement: S4 Fig — (TIF) [file pone.0117938.s004.tif]

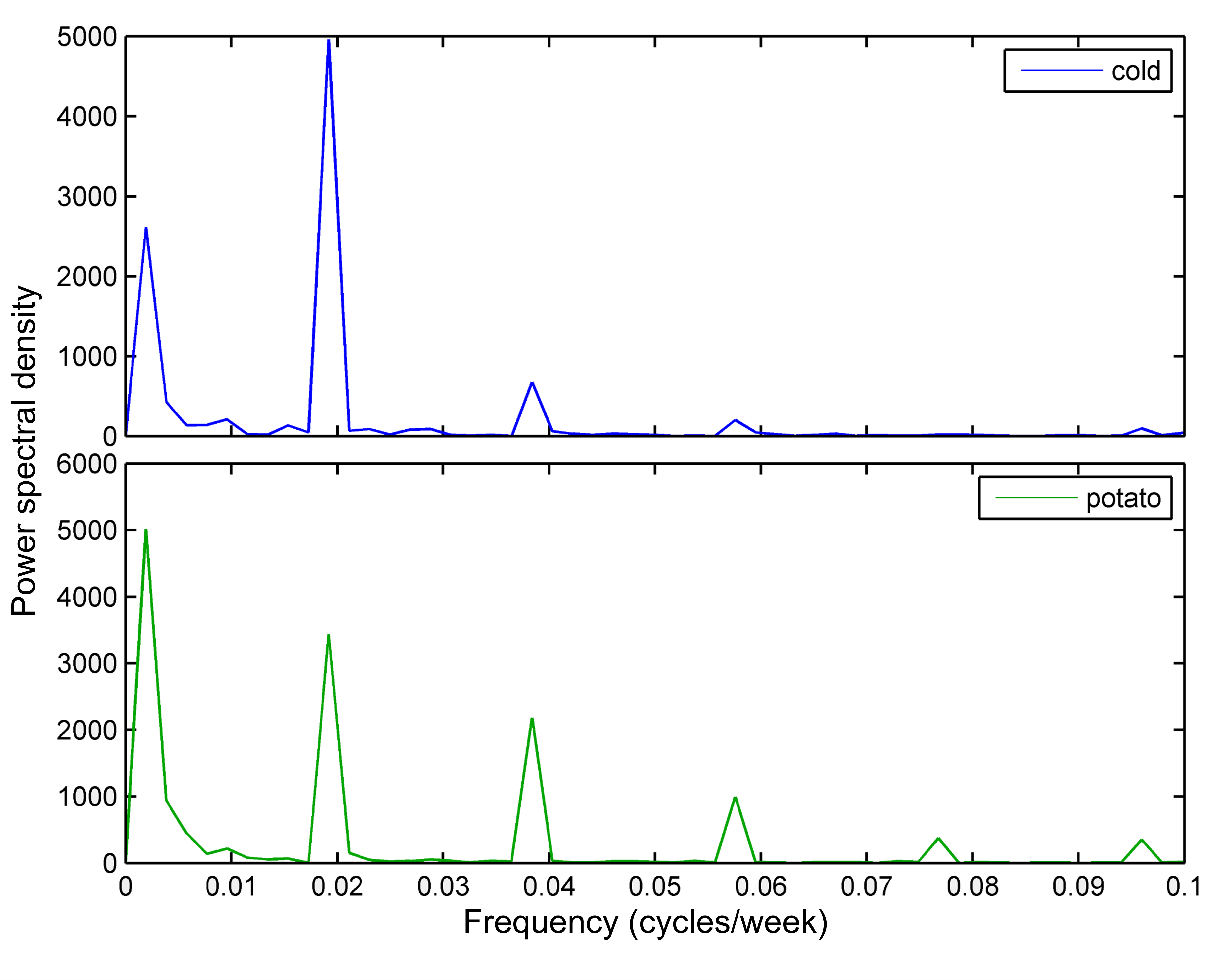

Supplement: S5 Fig — (TIF) [file pone.0117938.s005.tif]

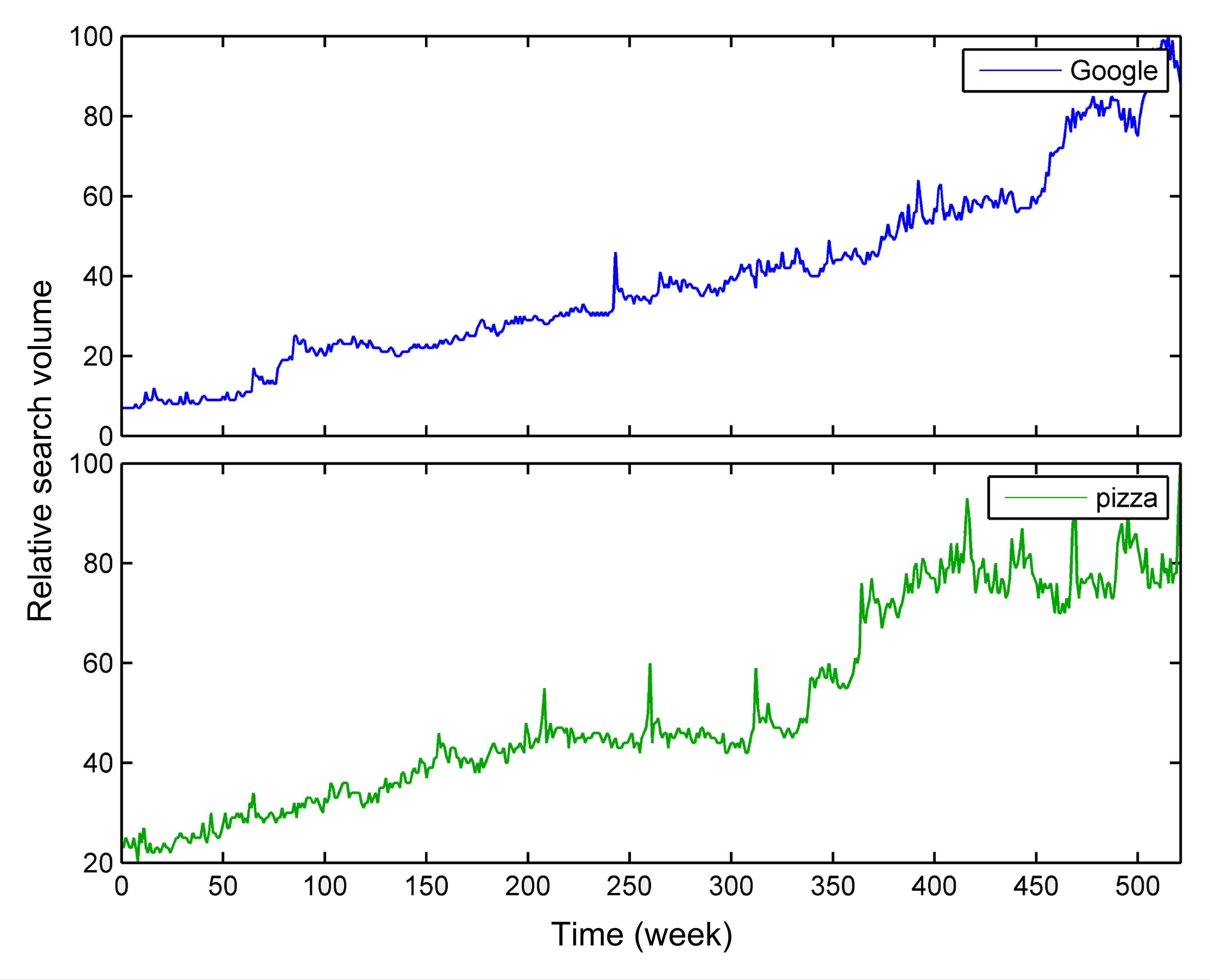

Supplement: S6 Fig — (TIF) [file pone.0117938.s006.tif]

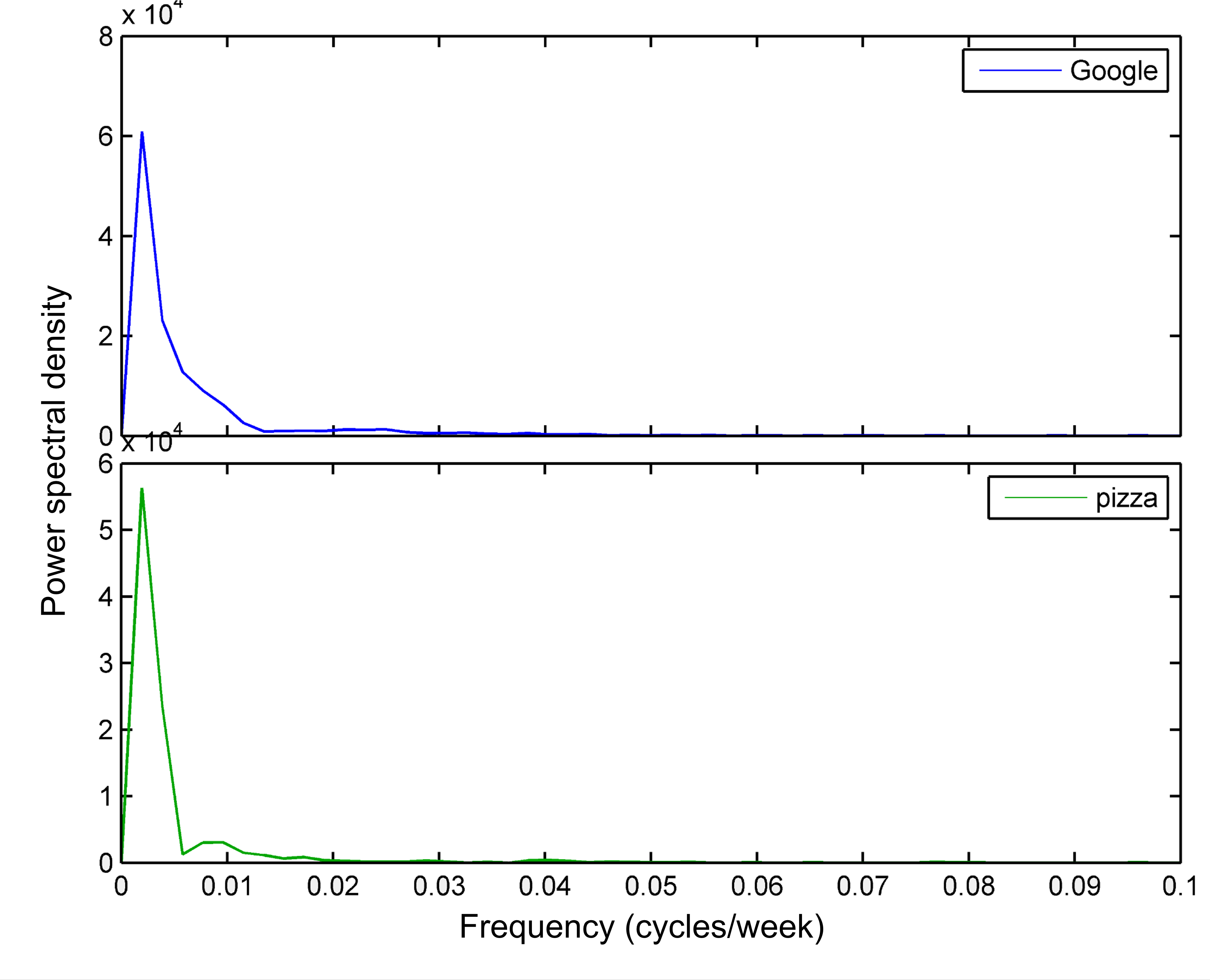

Supplement: S7 Fig — (TIF) [file pone.0117938.s007.tif]
