# Supplementary material for: Information Seeking Regarding Tobacco and Lung Cancer: Effects of Seasonality
Source: PLoS One. 2015 Mar 17;10(3):e0117938. doi: 10.1371/journal.pone.0117938 (PMC4364309; doi:10.1371/journal.pone.0117938)
Supplement: S2 Table — (DOCX) [file pone.0117938.s009.docx]

**Table S2.** The pairwise cross-correlations (at lag 0) of seasonal components of search trends regarding lung cancer.

|  | **Country** | | | | |
| --- | --- | --- | --- | --- | --- |
| **Country** | **US** | **Canada** | **UK** | **Australia** | **China** |
| **US** | 1.000 | 0.945 (0) | 0.438 (0) | 0.147 (0) | 0.045 (0) |
| **Canada** | -- | 1.000 | 0.540 (0) | -0.017 (0) | 0.137 (0) |
| **UK** | -- | -- | 1.000 | -0.578 (0) | 0.637 (0) |
| **Australia** | -- | -- | -- | 1.000 | -0.508 (0) |
| **China** | -- | -- | -- | -- | 1.000 |
