# Supplementary material for: Information Seeking Regarding Tobacco and Lung Cancer: Effects of Seasonality
Source: PLoS One. 2015 Mar 17;10(3):e0117938. doi: 10.1371/journal.pone.0117938 (PMC4364309; doi:10.1371/journal.pone.0117938)
Supplement: S3 Table — (DOCX) [file pone.0117938.s010.docx]

**Table S3.** Stationarity test of seasonal components of tobacco-related search trends.

|  | **Input parameter** | | **Output parameter** | |
| --- | --- | --- | --- | --- |
| **Country** | **lags** | **model** | **h** | **pValue** |
| US | 1 | ADR | 1 | 0.0010 |
| Canada | 1 | ADR | 1 | 0.0010 |
| UK | 1 | ADR | 1 | 0.0010 |
| Australia | 1 | ADR | 1 | 0.0010 |
| China | 1 | ADR | 1 | 0.0010 |

Augmented Dickey-Fuller test is used to test whether these trends are stationary. The test results indicate that there is enough evidence to suggest that these trends are stationary, autoregressive processes with a drift term.
